# Comparative Study of Denitrifying-MBBRs with Different Polyethylene Carriers for Advanced Nitrogen Removal of Real Reverse Osmosis Concentrate

**DOI:** 10.3390/ijerph17082667

**Published:** 2020-04-13

**Authors:** Tong Wang, Tong Wu, Haiyan Wang, Weiyang Dong, Yaqian Zhao, Zhaosheng Chu, Guokai Yan, Yang Chang

**Affiliations:** 1 School of Civil Engineering, Chang’an University, Xi’an 710061, China; wangt@chd.edu.cn (T.W.); wutong9408@163.com (T.W.); 2 State Key Laboratory of Environmental Criteria and Risk Assessment, Chinese Research Academy of Environmental Sciences, No. 8 Da Yang Fang, Anwai, Chaoyang District, Beijing 100012, China; docreat@163.com (W.D.); yangk@craes.org.cn (G.Y.); cy1100@126.com (Y.C.); 3 Engineering Center for Environmental Pollution Control, Chinese Research Academy of Environmental Sciences, No. 8 Da Yang Fang, Anwai, Chaoyang District, Beijing 100012, China; 4 National Engineering Laboratory for Lake Pollution Control and Ecological Restoration, Chinese Research Academy of Environmental Sciences, Beijing 100012, China; chuzs@craes.org.cn; 5 State Key Laboratory of Eco-Hydraulics in Northwest Arid Region, Xi’an University of Technology, Xi’an 710048, China; 6 UCD Dooge Center for Water Resources Research, School of Civil Engineering, University College Dublin, Belfield, Dublin 4, Ireland

**Keywords:** carriers, denitrification MBBR, nitrogen removal, real reverse osmosis concentrate

## Abstract

Nitrogen (N) remains a great challenge in wastewater treatment while attempts to remove N has continuously been a research point for decades. In this study, the long-term performance of four identical-shape denitrification MBBRs (moving bed biofilm reactors) with four different configurations of cylindrical polyethylene as carriers (Φ25 × 12, Φ25 × 4, Φ15 × 15, and Φ10 × 7 mm) for advanced N removal of real reverse osmosis concentrate was investigated in great detail. The N of the real concentrate can be effectively removed by denitrification MBBRs when the pH, temperature, hydraulic retention time (HRT), C/N ratio, and filling rate are 7.50–8.10, 24~26 °C, 12 hours, 6.6, and 50%, respectively. The results showed that the MBBR with the Φ15 × 15 poly-carrier had the best removal efficiency on NO_3_^-^–N (78.0 ± 15.8%), NO_2_^-^–N (43.79 ± 9.30%), NH_4_^+^–N (55.56 ± 22.28%), and TN (68.9 ± 12.4%). The highest biomass of 2.13 mg/g-carrier was in the Φ15 × 15 poly-carrier was compared with the other three carriers, while the genes of the Φ15 × 15 poly-carrier reactor were also the most abundant. Proteobacteria was the most abundant phylum in the system followed by Bacteroidetes and then Firmicutes. The entire experiment with various parameter examination supported that Φ15 × 15 poly-carrier MBBR was a promising system for N removal in high strength concentrate. Despite the lab-scale trial, the successful treatment of high strength real reverse osmosis concentrate demonstrated the reality of the treated effluent as possible reclaimed water, thus providing a good showcase of N-rich reverse osmosis concentrate purification in practical application.

## 1. Introduction

The water crisis caused by water pollution and water shortage has directly affected the sustainable development of the global economy and society [1]. Due to the lack of water resources, some water-deficient areas have used the effluent of wastewater treatment plants (WWTP) as a water source to produce high-quality reclaimed water [2]. One of the techniques to treat WWTP effluent is the low-pressure reverse osmosis membrane. However, it would produce a certain amount of reverse osmosis concentrate during the treatment process. Preliminary study showed that this type of reverse osmosis concentrate is different from conventional reverse osmosis concentrated water [3] and has the characteristics of high total nitrogen (TN) concentration and salinity, high NO_3_–^-^N/TN ratio, low C/N as well as poor biodegradability. Nitrogen (N) is one of the main elements of water eutrophication. Therefore, there is a need to remove the N in the reverse osmosis concentrate.

At present, the methods for treating reverse osmosis concentrate mainly include the Fenton reaction [4], advanced oxidation-nanofiltration [5], coagulation and ozone [6], coagulation sedimentation [7], membrane distillation and freeze crystallizer [8], electrodialysis with bipolar membranes [9], ultrasonic method [10], and biological treatment, which include the sequencing batch reactor (SBR) process [11], fluidized bed reactor (FBR) process [12], and anammox and moving bed biofilm reactor (MBBR) process [13]. Among the biological treatment processes, MBBR is an ideal wastewater treatment technique, combining the advantages of the activated sludge process and biofilm process. It has distinguishing advantages of high volume load, no clogging in the biological tank, strong impact resistance, low head loss, stable performance, reliable operation, high specific biomass activity, long service life, and no need of regular backwashing [14,15]. Although MBBR has been trialed for reverse osmosis concentrate treatment, it seems that they have been focused on micro-communities [13] and the biocarrier filling rate [16]. In addition, high rate N removal has been demonstrated by the MBBR, but there is still space to enhance the removal efficiency in MBBR [17]. 

The suspended carrier is the core of the MBBR process and provides a place for microorganisms to attach and grow. There are many types of suspended carries used in MBBR practice such as polyethylene (PE) [18,19], polypropylene (PP) [20], polyurethane (PU) [21], ceramsite [22], diatomite [23], and Poly(vinyl alcohol) hydrogels (PVA gel) [24]. Among these, PE has been widely used due to its good performance. Su et al. [18] used a lab denitrification MBBR with ф25 × 10 mm PE as the suspension carrier to treat Mn^2+^ containing wastewater. It was found that the system was suitable for nitrate removal in a concentration of 47.64 mg/L at a hydraulic retention time (HRT) of 11.96 h and pH of 5.21. The removal rates of N and Mn^2+^ were 77.44% and 61.28%, respectively. Zhang et al. [19] used PE (inclination angle 60°, filling rate 50%) as the MBBR carrier to treat ammonia nitrogen wastewater with 71.4 ± 26.9% being obtained. Yuan et al. [21] used denitrification-MBBR to treat the WWTP effluent with PE, PP, PU, and ceramsite as the carriers. In the stable operation stage, performances of denitrification, organic matter removal, biomass, and microbial characteristics on different carriers were compared. It was considered that PE was the most suitable carrier for denitrifying MBBR. It should be pointed out that in MBBR, the carrier is kept suspended by hydraulic force. After long term operation, the carrier could be separated via either sedimentation or flotation for washing or replacement. Recently, Feng et al. [25] and Zhao et al. [26] successfully developed an approach of sulfidization-flotation of a carrier (smithsonite) in the presence of ammonia and explored the mechanism of ammonia modified mineral surface for enhanced flotation behavior. It is reasonable to believe that this approach may be useful for the N-rich wastewater treatment in the MBBR process. 

So far, MBBR has been widely used to treat various kinds of wastewaters such as paper mill wastewater [27], coal gasification wastewater [28], pharmaceutical industry wastewater [29,30], fish farm wastewater [31], and reverse osmosis concentrate [13,16,32], while the denitrification performance of the systems was affected by the carrier material, configuration, and pore space structure as well as the surface morphology of the biofilm [33]. However, there have been no reports on the deep denitrification of reverse osmosis concentrate by denitrifying MBBR with different configurations of polyethylene carriers, which forms the basis of the current study.

In this paper, four different configurations of polyethylene were used as denitrifying MBBR carriers with a filling rate of 50% to explore the advanced N removal of real reverse osmosis concentrate. The removal efficiency of ammonia nitrogen (NH_4_^+^–N), nitrite (NO_2_–^-^N), nitrate (NO_3_–^-^N), and total nitrogen (TN) were compared. The biofilm microbial characteristics of each carrier were examined by scanning electron microscopy (SEM), quantitative polymerase chain reaction (Q-PCR), and high throughput technology. Accordingly, the optimal polyethylene carrier with the best size was determined. Through the designed comparative study, it is expected to that the treatment mechanism will be revealed, especially the role of the biocarrier, which is considered to be the key factor to ensure the treatment efficiency, and thus provide insight into the overall process for high strength N treatment from a real reverse osmosis concentrate.

## 2. Materials and Methods 

### 2.1. Reactor Set-Up and Operation

**Figure 1:**
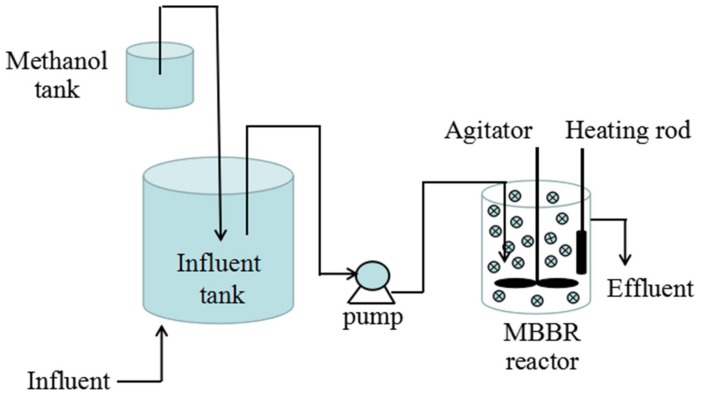
Schematic description of the moving bed biofilm reactor (MBBR) experimental setup.

The four reactors/MBBRs were made of Plexiglass cylinders (186 mm in diameter and 230 mm in height) with 6.24 L anoxic zone. As schematically shown in Figure 1, agitator was used to mix the carriers and sludge inside. Temperature was controlled at 25 ± 1 °C by heating rod. Influent was continuously fed to the reactors by peristaltic pumps (BT100-1L, Baoding Lange Constant Pump Co. Ltd.). The reactors were inoculated by activated sludge, which was collected from the anoxic tank of Beijing Xiao Jia He Wastewater Treatment Plant (WWTP) (located in Beijing city, China, with the geographical coordinates of 39°54′49.7448′′N and 116°21′49.0500′′ E). The mixed liquid suspended solids (MLSS), mixed liquor volatile suspended solids (MLVSS), MLVSS/MLSS, settling velocity (SV), and sludge volume index (SVI) of the inoculation were 6300 mg/L, 2898 mg/L, 0.46, 57%, and 90.48 mL/g, respectively. Three liters of activated sludge was dosed with 3.24 L reverse osmosis concentrate in each reactor. 

The characteristics of the four PE cylinders are shown in Table 1. The filling ratio of the carriers was set at 50% in this study [19,34]. During the system operation, NH_4_^+^–N, NO_2_^-^–N, NO_3_^-^–N, TN, and pH of the influent and effluent were monitored every two days. HRT and pH were controlled at 12 h and 7.5–8.1 [16], respectively, and agitation speed was adjusted to 60 rpm. A total of 45.5 mg/L methanol was added to maintain the COD/TN ratio at 6.6, while the original ratio was <4.6. 

### 2.2. The Characteristic of Reverse Osmosis Concentrate

Reverse osmosis concentrate produced by the low pressure reverse osmosis unit for the high-quality water reclamation was collected from Beijing Cui Hu WWTP. The concentrate had the characteristics of high salinity, low C/N ratio, and high TN concentration with NO_3_^-^–N of more than 70%. It was used as the MBBR influent with the addition of methanol as the carbon source. The influent characteristics are listed in Table 2.

### 2.3. Analyses of Water Quality

Chemical oxygen demand (COD) and NH_4_^+^–N were analyzed according to the Chinese Standard Methods as used by Sun et al. [35]. TN was determined by a TOC-V_CPH_ total organic carbon (TOC) analyzer (Shimadzu, Kyoto, Japan), while NO_2_^−^–N and NO_3_^−^–N were analyzed by ion chromatography (DIONEX ICS-1000, Sunnyvale, California, USA). Samples for NO_2_^−^–N, NH_4_^+^–N, and NO_3_^−^–N were pretreated by a 0.45 μm membrane filter.

### 2.4. Microbial and Molecular Biology Analysis

The appropriate amounts of carrier samples were collected from the MBBRs on day 224 when each MBBR performed most efficiently. After collection, they were immersed in 20 mL of a 1 M NaOH solution in a water bath at 80 °C for 30 min, followed by one minute ultrasound (at 100 W), and 30 s vortex treatment before biomass analysis [36]. SEM (JSM-5800, Japan JEOL, Tokyo, Japan) observations and flow cytometry (BD Accuri C6, San Jose, CA, USA) were used for the analysis of the biofilms and quantitated the cells on the plastic carriers, respectively. The carriers were shaken in a shocking incubator for 24 hours to obtain the falling biofilm. The carries were then washed three times with deionized water to rinse off the remaining biofilm. Procedures of the SEM observations were done following the description of Liu et al. [6]. 

#### 2.4.1. Quantitative Polymerase Chain Reaction (Q-PCR) Analysis

Q-PCR technology is widely used to investigate the gene richness of the total bacteria and the richness of denitrifying bacteria in the biofilm of MBBRs [21]. In this study, the bacterial 16S rRNA gene, 16S Archaea (for total bacteria), and the narG, nirS, nirK genes (for denitrifiers) were applied as primers [37,38]. The detailed denitrification primer design is shown in Table 3. DNA was extracted using an UltraClean DNA extraction kit (Mobio Laboratories, Carlsbad, CA, USA). The PCR operating conditions were consistent with those used by Scala and Kerkho [39]. Each sample was performed three times and the average datum was reported.

#### 2.4.2. High Throughput Analysis

In order to remove low quality and chimeric sequences, the original sequencing reads were processed using Mothur software [43]. All datasets were then diluted to the 253501 sequence to achieve the same sequencing depth. The community structure of samples at different phylum level was observed through statistical analysis. 

## 3. Results and Discussion

### 3.1. Nitrogen Removal

#### 3.1.1. NO_3_^-^–N and TN Removal

**Figure 2:**
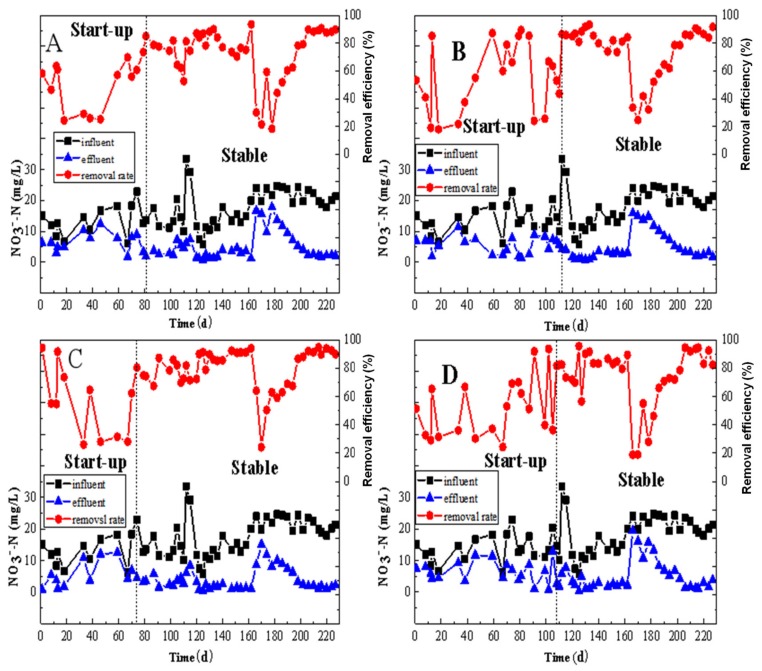
The removal performance of NO_3_^-^–N by MBBRs. (**A**): the MBBR A; (**B**): the MBBR B; (**C**): the MBBR C; and (**D**): the MBBR D.

Figure 2 displayed the removal efficiency of NO_3_^-^–N from the MBBRs with four different configurations of cylindrical polyethylene (Φ25 × 12, Φ25 × 4, Φ15 × 15, and Φ10 × 7 mm). At the beginning of operation, the removal rate of NO_3_^-^–N in MBBRs varied with the influent fluctuation and the MBBRs were unstable. This is normal as the MBBR needs time to establish the biological environment, especially the time required to form the biofilm on the carriers inside the MBBR. It is well accepted that the operation time (days) for the reactor to reach the relative stable treatment efficiency is considered as the start-up period. The concentration of the NO_3_^-^–N in reactor A (MBBR A) during the start-up period (1–79 days) was 13.43 ± 4.71mg/L, while its removal efficiency was 50.1 ± 17.2%. Reactor B was successfully started after day 110. The NO_3_^-^–N concentration in the start-up period was 13.65 ± 4.22 mg/L, while the removal efficiency was 55.3 ± 24.0%. The effluent NO_3_^-^–N concentration of the reactor B was about 1.06 mg/L, lower than that of reactor A. Reactor C was the fastest reactor out of the four to become stable as it started successfully after 70 days of operation (Figure 2C). The NO_3_^-^–N concentration and its removal efficiency in the start-up period were 12.64 ± 4.18mg/L and 55.5 ± 23.8%, respectively. After 105 days of operation, reactor D was successfully started. The influent NO_3_^-^–N concentration was 13.79 ± 4.35 mg/L, while the removal efficiency of 51.2 ± 20.3% was obtained. During the start-up period of four MBBRs, the removal rate of NO_3_^-^–N in reactor A was the lowest, followed by reactor D. The removal rates of NO_3_^-^–N in the two reactors of B and C were basically the same. In the four denitrified MBBRs, reactor C started the fastest and reactor B started the slowest.

After the start-up stage, the removal efficiencies of NO_3_^-^–N in all of the reactors showed a trend of first stabilization, then decrease, increase, and then finally re-stabilization. The reason for the decrease was the power failure of the laboratory during the trial period. The microbial activity in each reactor was therefore affected. There was an adaptive stage in the microorganisms after the reactor was resumed. The activity of the microorganisms seemed to increase continuously, and then the stable state was reached once again.

The overall influent NO_3_^-^–N concentrations of reactors A, B, C, and D were 17.77 ± 5.98 mg/L, 18.71 ± 6.13 mg/L, 17.77 ± 5.94 mg/L, and 18.34 ± 6.16 mg/L, respectively, while the corresponding removal efficiencies were 72.5 ± 19.0%, 75.0 ± 18.6%, 78.0 ± 15.8%, and 74.8 ± 20.1%, respectively. The removal efficiency of NO_3_^-^–N of reactor A was the lowest, which is believed to be linked with the overall surface areas in reactor A. The removal efficiency of reactor C was the highest, while those in reactors B and D were basically the same, with only a difference of 0.2%. Reactors A, B, and C were restored to a stable state from day 198, eight days later than reactor D.

The NO_3_^-^–N loadings of the four reactors were 0.074 g/m^2^∙d, 0.056 g/m^2^∙d, 0.1300 g/m^2^∙d, and 0.067 g/m^2^∙d, respectively. The average denitrification rate of the reactor C was 0.1014 g/m^2^∙d. It was 0.0478 g/m^2^∙d, 0.060 g/m^2^∙d, and 0.0513 g/m^2^∙d higher than that of reactors A, B, and D, respectively, suggesting that reactor C was most effective than any other reactors for NO_3_^-^–N removal in denitrification MBBRs. The possible reason is that reactor C provided large internal surfaces on which the reactor could effectively prevent the slow-growing bacteria from being washed away, and thus facilitate biofilm attachment [44], whereas the others three reactors had a relatively small internal surface.

**Figure 3:**
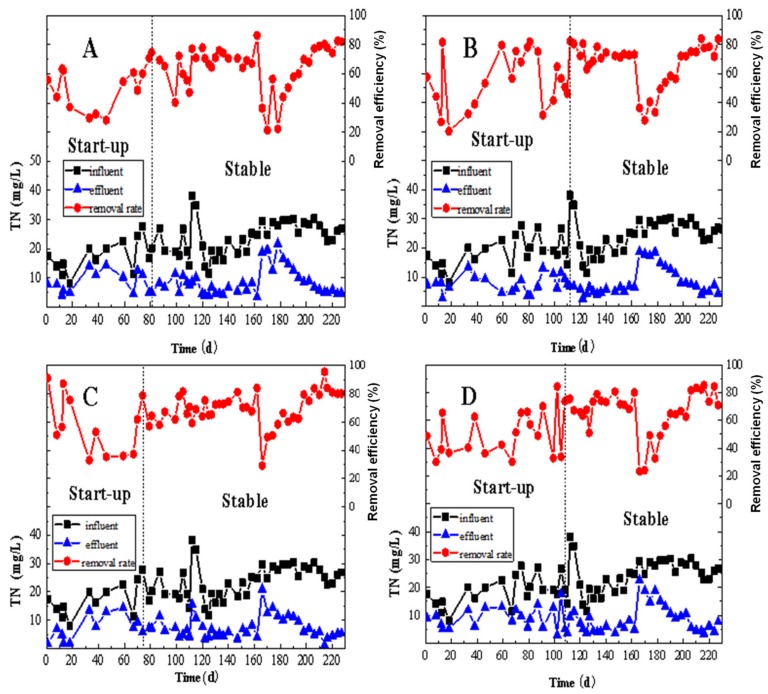
The removal performance of the total nitrogen (TN) by MBBRs: (**A**): the MBBR A; (**B**): the MBBR B; (**C**): the MBBR C; and (**D**): the MBBR D.

The performance of TN removal in the four MBBRs is illustrated in Figure 3. At the beginning of the operation, due to the new environment of activated sludge and lower activity of the microorganisms, the removal efficiency of TN was low. With the operation of the reactors, microorganisms were gradually adapted to the internal environment and TN removal efficiency also increased. After the microorganisms completely adapted, the reactors began to enter a stable period. Reactor A became stable from day 81, while reactors B, C, and D were stable from days 112, 74, and 108, respectively. 

During the start-up period, the influent TN concentrations of reactors A, B, C, and D were 17.32 ± 5.42 mg/L, 18.52 ± 5.18 mg/L, 16.41 ± 4.88 mg/L, and 18.70 ± 5.36 mg/L, respectively. Accordingly, the removal efficiencies were 49.9 ± 13.7%, 55.3 ± 18.7%, 56.1 ± 19.9%, and 49.6 ± 15.4%, respectively. The TN removal efficiencies in reactors A and D were basically the same, both were less than 50%, with the difference between them of only about 0.3%. However, reactor A showed a slight fluctuation. The removal rate of TN in reactor C was higher than that in reactor B. 

When the system became stable, the influent TN concentrations of the four denitrifying MBBRs of A, B, C, and D were 23.86 ± 5.79 mg/L, 24.70 ± 5.85 mg/L, 23.78 ± 5.78 mg/L, and 24.24 ± 6.00 mg/L, respectively, and a considerable increase (by average 36.2%) of the influent TN concentrations was observed. This is because the season’s operation of the real reverse osmosis unit and the resultant reverse osmosis concentrate. In the stable stage, the TN removal efficiency was in the pattern of C = 68.9 ± 12.4% > B = 67.01 ± 14.8% > D = 66.6 ± 15.6% > A = 65.0 ± 15.2%. They were quite similar, but reactor C was the highest, indicating that reactor C had the best performance. On day 166, it was found that the total inlet pipe was broken and leakage occurred, which resulted in the abnormal intake water of each reactor and affected the normal operation of the reactors. Therefore, the TN removal was affected and this is reflected in Figure 3. Reactors B and C were restored to a stable state on day 198, while the recovery time of reactors A and D was relatively long, from day 206.

#### 3.1.2. NO_2_^-^–N and NH_4_^+^–N Removal

**Figure 4:**
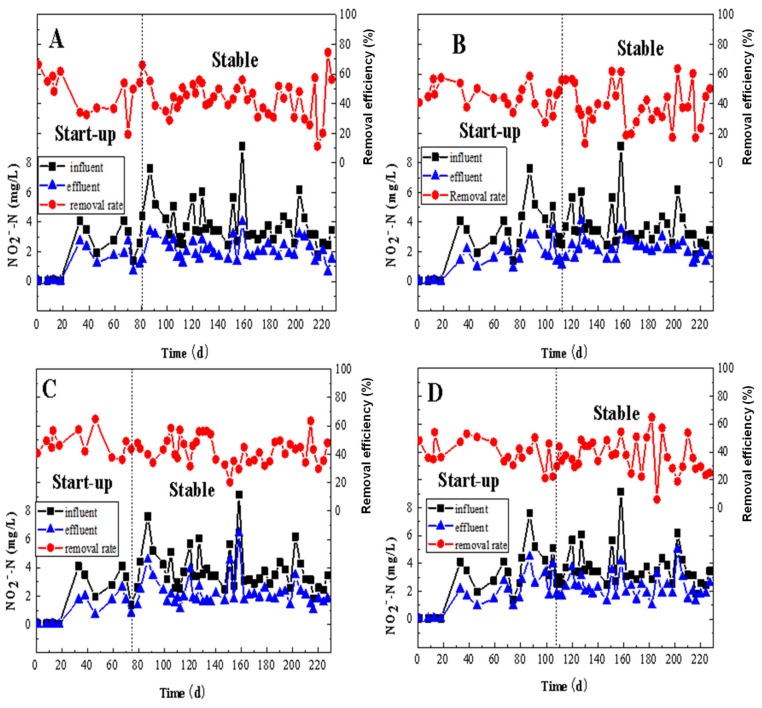
The removal performance of NO_2_^-^–N by MBBRs: (**A**): the MBBR A; (**B**): the MBBR B; (**C**): the MBBR C; and (**D**): the MBBR D.

At the beginning of the operation, the removal rate of NO_2_^-^–N in MBBRs varied with the changes in influent concentration (Figure 4). Under the influent NO_2_^-^–N concentration of 3.38 ± 1.73 mg/L, the removal efficiency of NO_2_^-^–N in reactors A, B, C, and D was 44.28 ± 12.52%, 41.39 ± 12.53%, 43.79 ± 9.30%, and 38.79 ± 9.30%, respectively. The effluent NO_2_^-^–N concentrations were 1.88 ± 0.89 mg/L, 1.92 ± 0.89 mg/L, 1.94 ± 1.15 mg/L, and 2.08 ± 1.09 mg/L in reactors A, B, C, and D, respectively. The removal efficiencies in reactors A, B, and C were basically the same, while that of reactor D was lower than in the other three reactors. During the whole operation period, the highest removal efficiency was 74.69% in reactor A on day 224, while the lowest was 11.22% on day 216. The highest removal efficiency of reactor B was 63.55%, which was reached on day 202, while on day 130, the lowest removal efficiency of 13.15% was recorded. On day 46 of operation, reactor C reached the highest removal efficiency of 64.84%. Its lowest removal efficiency of 20.15% was recorded on day 151. Regarding reactor D, the highest and lowest removal efficiencies were 64.86% and 5.87%, respectively, which were reached on days 182 and 186, respectively. Therefore, NO_2_–^-^N removal in the whole MBBR operation seemed good when compared with other similar studies in the literature [31,32,44]. There are two possible reasons. One is that during the operation of the reactors, air entered due to improper sealing, forming a local aerobic environment in the reactors and nitrification occurred. The other is that the original NO_2_^-^–N reacts with NH_4_^+^–N under the anaerobic environment inside the carriers. There, the co-existence of denitrification and anammox for simultaneous nitrogen [43] was reported, although there was no identification of anammox being conducted in this study. Anaerobic ammonia oxidation takes ammonia as the electron donor and nitrite as the electron acceptor under the anaerobic condition, and converts ammonia and nitrite into nitrogen under the reaction of NH_4_^+^ + NO_2_^-^ → N_2_ + 2H_2_O [45].

**Figure 5:**
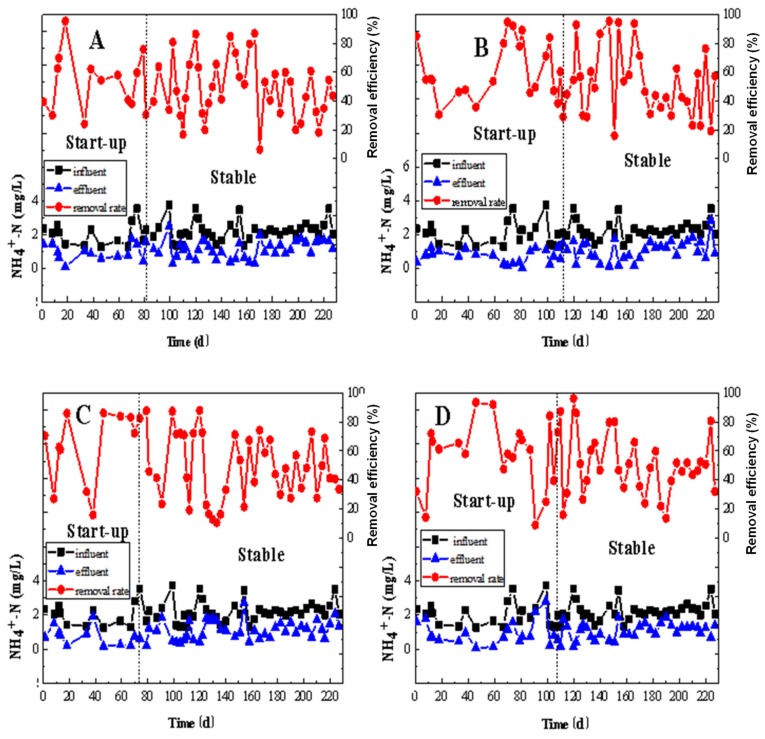
The removal performance of NH_4_^+^–N by MBBRs: (**A**): the MBBR A; (**B**): the MBBR B; (**C**): the MBBR C; and (**D**): the MBBR D.

The performance of the NH_4_^+^–N removal efficiency of the MBBRs with different configurations of PE carriers during the entire operation is visualized in Figure 5. At the beginning of the operation, the removal efficiency of NH_4_^+^–N in each MBBR was quite different. This was because of the difference in the initial biofilm formation strategies, which contributed to the higher amount of attached biomass in reactor B than in any of the other three reactors (Figure 5). The NH_4_^+^–N removal efficiency of reactors A, B, C, and D was 49.53 ± 20.37%, 51.83 ± 23.52%, 55.56 ± 22.28%, and 53.03 ± 21.83%, respectively, when the influent NH_4_^+^–N concentration was 2.16 ± 0.60 mg/L. The effluent NH_4_^+^–N concentration was 1.09 ± 0.50 mg/L, 1.03 ± 0.56 mg/L, 0.93 ± 0.55 mg/L, and 1.02 ± 0.56 mg/L, respectively. The removal efficiencies of NH_4_^+^–N in reactors B and C were basically the same. Reactor A was lower than that in the other three reactors. The removal efficiency of NH_4_^+^–N in reactor C was significantly higher than that in the other three reactors. Among them, the highest removal efficiency of reactor A was 95.65%, which was reached on day 18. On day 170, the removal efficiency reached the lowest at 6.24%. The highest removal efficiency of reactor B was 87.93% recorded on day 120, while its lowest removal efficiency of 10.66% was occurred on day 133. Reactor C had the highest removal efficiency of 95.55% on day 147, while the lowest removal efficiency was 15.77% on day 151. Reactor D achieved its highest removal efficiency of 96.05% on day 120 of operation. On day 91, the lowest removal efficiency of 8.93% was recorded. During the whole operation, the NH_4_^+^–N removal in the denitrification MBBRs was generally about 50%, not as high as expected, which may indicate some other processes such as partial nitrification occurrence in the reactors.

### 3.2. Microbial Characteristics of Moving Bed Biofilm Reactor (MBBRs)

#### 3.2.1. Biomass Analysis

Adabju [46] reported that the biomass of the filler was 0.95–5.0 mg/g in a stable operation of a MBBR reactor with polyethylene as the suspension filler. The biomass of the current study was between 1.74 and 2.13 mg/g or 8.94–13.76g∙SS/m^2^, which is consistent with Adabju [46]. With a filling ratio of the suspended carriers of 30% and influent loading rate of 1.43–2.13 g N/(m^2^·day) at 11–17 °C, Stinson et al. [47] found that the biomass on the suspended carriers was 12–22 g∙ SS/m^2^, which was slightly higher than the biomass in this study. There are three possible reasons. First, the concentration and load of NO_3_^-^–N in the influent of this study were low. Stinson et al. [44] believed that when the concentration of NO_3_^-^–N was low, the biomass in the MBBR would be relatively small. Second, during the entire experimental operation, the temperature of this study was around 25 °C, while Shrestha et al. [48] and Peric et al. [49] claimed that the biomass would decrease with the decreasing temperature. At low temperature, thick and numerous biofilms can be formed [50]. Finally, the filling ratio of the suspended carriers was 50% in this study. It has been found that high carrier filling rate could increase the risk of biofilm detachment from the media [51]. 

By comparing the biomass in this study, it was found that the biomass of the four carriers in the corresponding MBBRs was C (2.13 mg/g) > A (1.85 mg/g) > B (1.83 mg/g) > D (1.74 mg/g) when the operation was stable. The biomass of poly-carries in C was the highest, which may be due to the high porosity of more than 90%, higher than the other three poly-carriers (porosity >85%) in this study. In addition, polyethylene in C had a higher geometric height than the other three and provided a bigger inter-surface, which can effectively reduce the shedding of biofilm under water flow shear [14,42]. This is also the reason why the removal rate of NO_3_^-^–N, NO_2_^-^–N, NH_4_^+^–N, and TN in MBBR C was higher than that of other three MBBRs during the whole trial period. The biomass in A and B was not much different, probably because the porosity was the same, while the pore numbers inside the filler was not much different (40 and 64, respectively). MBBR D had the smallest biomass, probably because of its minimum number of holes in the carries.

#### 3.2.2. Scanning Electron Microscopy (SEM) Observation

**Figure 6:**
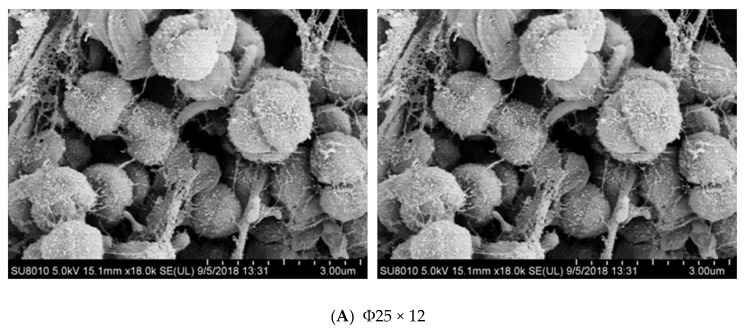

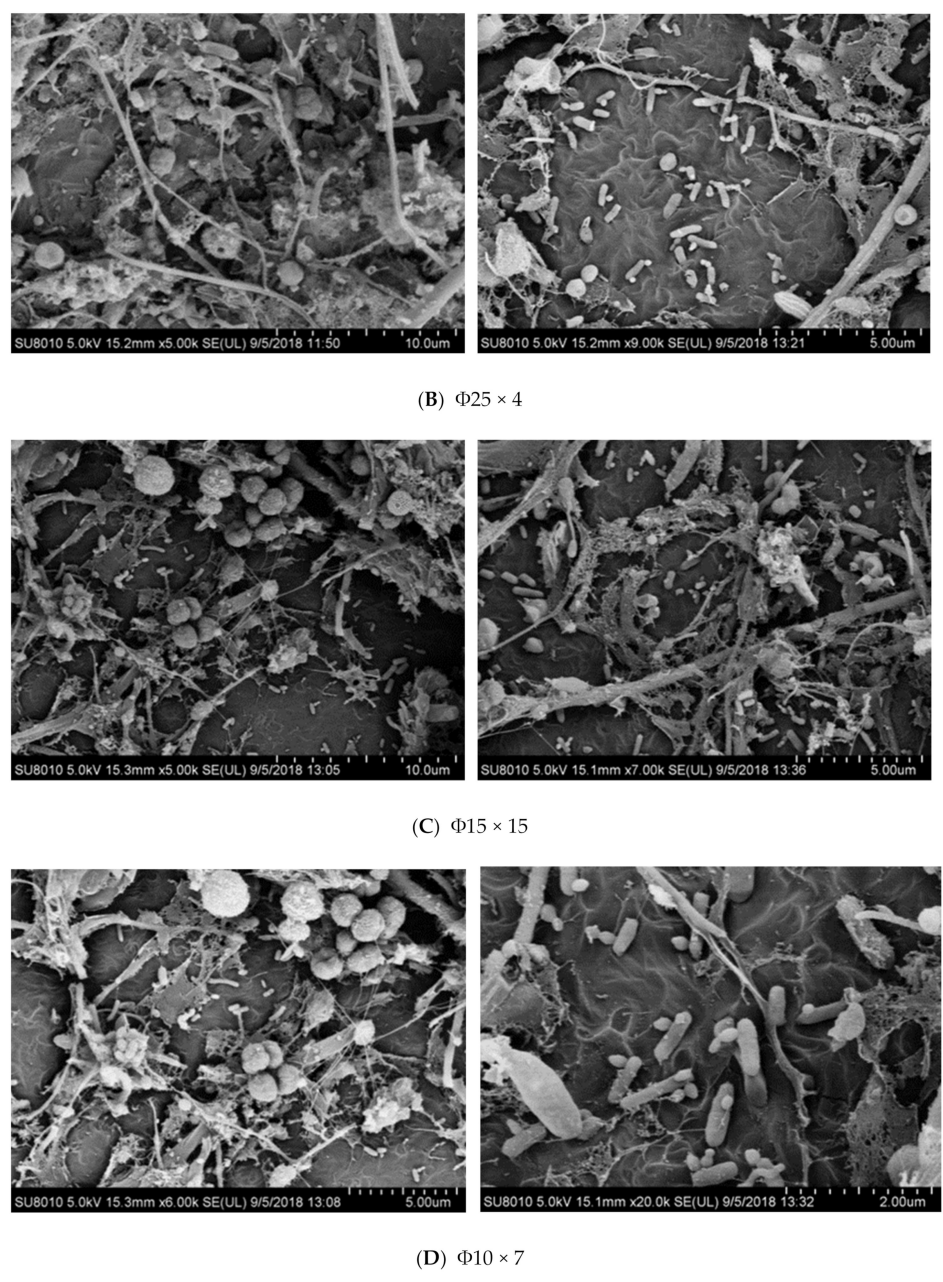
Scanning electron microscopy image of four different polyethylene carriers: (**A**): the MBBR A; (**B**): the MBBR B; (**C**): the MBBR C; and (**D**): the MBBR D.

SEM observation of the four different poly-carriers is shown in Figure 6. It appears that the microorganisms attached to the carriers were mainly cocci, bacilli, and filamentous bacteria with the cocci and rod-shaped bacteria on the poly-carriers in B and C being more obvious. There were fewer cocci and rod-shaped bacteria on the poly-filler in D. In addition, cocci, bacilli, and filamentous bacteria all co-exist in the four poly-carriers, which can make the biofilm more compact and more stable. The biofilm in D is relatively sparse, not as dense as the other three suspension fillers. Overall, the cocci are clustered together, while the rod-shaped bacteria are more dispersed and more viscous substances. The reason may be that bacteria in biofilms undergo completely different microenvironments [52]. The four carriers were rich in microbial species, leading to the system being stable. The denitrifying bacteria were mostly cocci and bacilli, which are consistent with the existing study [53]. Indeed, the microorganisms on the poly-carriers in C were the most abundant and compact.

#### 3.2.3. Q-PCR Analysis

Table 4 shows the gene richness of 16S bacteria, 16s archaea, nirK, nirS, and narG in the four reactors. The 16s bacteria and narG gene richness in the four reactors were similar, but these two genes were more abundant in reactor C. The number of 16s archaeal genes in reactor C was 1.45 × 10^11^ copies/g-SS, which was about one order higher than that of reactors A and B. The 16s archaea in reactor D was the least, 9.44 × 10^9^ copies/g-SS. The abundance of nirK gene in reactors A, C, and D was basically the same, which is about one order of magnitude lower than that of reactor B. The nirS gene was the most in reactor C, which was 5.53 × 10^10^ copies/g-SS, followed by reactor A (1.40 × 10^10^ copies/g-SS), then reactor D (9.75 × 10^9^ copies/g-SS) and reactor B (2.98 × 10^9^ copies/g-SS). Overall, the gene of the biofilm in reactor C was the most abundant, while the biofilm of reactor A had the worst gene richness.

#### 3.2.4. High Throughput Analysis

**Figure 7:**
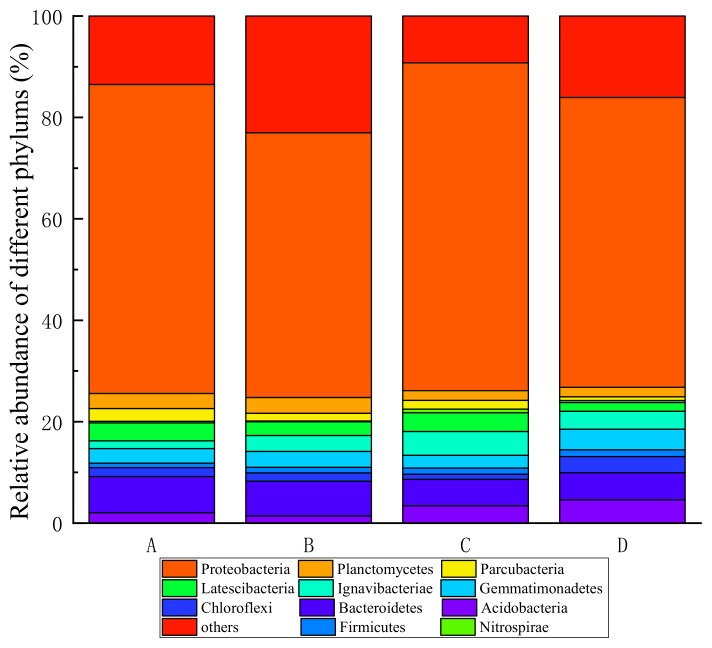
The microbial community in the phylum level.

To investigate the microbial community during the operation of denitrification MBBRs, the 16S rRNA gene based high-throughput sequencing technology was applied to analyze the biofilm samples and the results are illustrated in Figure 7. Obviously, the most abundant phylum in the four samples was *Proteobacteria*, which accounted for about 60.93% (reactor A), 52.20% (reactor B), 64.67% (reactor C), and 58.29% (reactor D) of the whole sequences. These were in accordance with the statement that *Proteobacteria* accounted for 36–65% of total effective bacteria in WWTPs [54]. This indicates that *Proteobacteria* attached to the carrier is more adaptable to the environment in the denitrifying MBBR reactor than other bacteria. As many reports have shown, most denitrifying bacteria belong to phyla *Proteobacteria* [55]. The *Proteobacteria* community was Gram-negative bacteria and contained large number of denitrificans [56], whose outer surface lipopolysaccharides could assist with their attachment to the carrier surface [57]. In addition, *Bacteroidetes* and *Firmicutes* also play a very important role in denitrification [11,58]. *Bacteroidetes* accounted for about 7.12–10.82% of the total bacteria, which was the second highest frequency phylum. Bassin et al. [44] showed that *Bacteroidetes* accounted for about 13% of the sequence reads in an anoxic moving-bed biofilm reactor. The minor differences may be caused by the dependence of bacteria in the reactor on the influent water [59]. *Firmicutes* was the second most frequency phylum, which accounted for about 4.11%–5.38% of the total bacteria. *Bacteroidetes* and *Firmicutes* have been identified as denitrifcans [60] and have been found as dominant phyla in many lab-scale denitrification reactors [51,61,62] and in two full-scale MBBRs with suspended polyethylene carriers [57]. Autotrophic bacteria accounted for a particularly small proportion of total bacteria (0.31%, 0.14%, 0.21%, 0.39% and 1.73%, 1.64%, 1.02%, 1.23%, respectively, for the four reactors). The results of this study were similar to those of other sewage treatment systems on taxonomic community [63].

## 4. Conclusions

The study demonstrated the effectiveness of using four different configurations (Φ25 × 12, Φ25 × 4, Φ15 × 15, and Φ10 × 7 mm) of cylindrical polyethylene as carriers in denitrification MBBRs for N removal from a real reverse osmosis concentrate. The results of the stabilization phase were as follows:

- The MBBR C with Φ15 × 15 polyethylene as the carrier had the best removal performance on NO_3_^-^–N, NO_2_^-^–N, NH_4_^+^–N, and TN in real reverse osmosis concentrate, which was 78.0 ± 15.8%, 43.79 ± 9.30%, 55.56 ± 22.28%, and 68.9 ± 12.4%, respectively.
- The MBBR C had most abundant microorganisms (2.13 mg/g-carrier). SEM observations showed that many short rod bacteria were attached to the suspended filler. These were similar in shape and size to some denitrificants. The gene of the biofilm on reactor C was the most abundant, while the biofilm of reactor A had the worst gene richness. From high throughput analysis, *Proteobacteria* are the most abundant phylum, followed by *Bacteroidetes*, then *Firmicutes*.
- According to the N removal efficiency and biomass as well as the microbial characteristic of four poly-carriers, the Φ15 × 15 poly-filler in reactor C was recommended as the best MBBR carrier for denitrification.

Overall, this study provides a ready to use technique that is directly linked with industrial application for reverse osmosis concentrate and other similar wastewaters.

## Figures and Tables

**Table 1:** Parameters of different carriers in the MBBRs.

Reactor	Configurations (mm)	Density (g/cm^3^)	Specific Surface Area (m^2^/m^3^)	Porosity (%)	Number of Pores
A	Φ25 × 12	0.96–0.98	>900	>85%	40
B	Φ25 × 4	0.96–0.98	>500	>90%	19
C	Φ15 × 15	0.96–0.98	>1200	>85%	64
D	Φ10 × 7	0.96–0.98	>1000	>85%	5

**Table 2:** Influent characteristics.

Operation Time (d)	C/N	Salinity (‰)	TN (mg/L)	NH_4_^+^–N (mg/L)	NO_3_^-^–N (mg/L)	NO_2_^-^–N (mg/L)
1~227days	6.6	0.5 ± 0.2	22.3 ± 6.4	2.2 ± 0.6	16.7 ± 5.9	3.8 ± 1.7

**Table 3:** Primer pair used in the denitrifying MBBR assay.

Gene	Primer	Sequence(5′-3′)	Reference
16s rRNA	16s-f	ATGGCTGTCGTCAGCT	[40]
	16S-R	ACGGGGCGGTGTGTAC	
16S Archaea	519F	CAGCMGCCGCGGTAA	[37]
	Arch915R	GTGCTCCCCCGCCAATTCCT	
narG	narG-F	TCGCCSATYCCGGCSATGTC	[40]
	narG-R	GAGTTGTACCAGTCRGCSGAYTCSG	
nirS	cd3AF	GTSAACGTSAAGGARACSGG	[41]
	R3cd	GASTTCGGRTGSGTCTTGA	
nirK	nirK1F	GGMATGGTKCCSTGGCA	[42]
	nirK5R	GCCTCGATCAGRTTRTGG	

**Table 4:** Biofilm Q-PCR analysis.

Unit (copies/g-SS)	A (Φ25 × 12)	B (Φ25 × 4)	C (Φ15×15)	D (Φ10 × 7)
16S bacteria	3.51 × 1010	1.25 × 1010	9.34 × 1010	1.6 × 1010
16S archaea	6.10 × 1010	2.05 × 1010	1.45 × 1011	9.44 × 109
nirK	2.06 × 105	2.37 × 106	3.70 × 105	2.24 × 105
nirS	1.40 × 1010	2.98 × 109	5.53 × 1010	9.75 × 109
narG	2.03 × 108	4.47 × 108	5.21 × 108	1.25 × 108
